# Th1/Th17-Related Cytokines and Chemokines and Their Implications in the Pathogenesis of Pemphigus Vulgaris

**DOI:** 10.1155/2017/7151285

**Published:** 2017-02-22

**Authors:** Rodolfo Pessato Timoteo, Marcos Vinicius da Silva, Camila Botelho Miguel, Djalma Alexandre Alves Silva, Jonatas Da Silva Catarino, Virmondes Rodrigues Junior, Helioswilton Sales-Campos, Carlo Jose Freire Oliveira

**Affiliations:** Laboratory of Immunology, Federal University of Triângulo Mineiro, Uberaba, MG, Brazil

## Abstract

Pemphigus vulgaris (PV) is an autoimmune disease characterized by the presence of IgG autoantibodies against desmoglein-3. Despite the variety of findings, the chemokine and cytokine profiles that characterize the immune response in the disease are still poorly explored. Thus, 20 PV patients and 20 controls were grouped according to gender, ethnicity, place of residence, and clinical parameters of the disease. Then, the levels of chemokines and of Th1/Th2/Th17/Treg/Th9/Th22-related cytokines were assessed in the serum. PV patients had higher levels of inflammatory Th1/Th17 cytokines (IFN-*γ*, IL-17, and IL-23), as well as higher levels of CXCL8 and reduced levels of Th1/Th2-related chemokines (IP-10 and CCL11). However, no differences in the levels of IL-2, IL-6, TNF-*α*, IL-1*β*, IL-4, IL-9, IL-12, TGF-*β*, IL-33, MCP-1, RANTES, and MIP-1*α* were found between PV patients and their control counterparts. Furthermore, PV patients with skin lesions had higher serum levels of IL-6 and CXCL8 when compared to PV patients without lesions. Taken together, our findings describe the role of cytokines and chemokines associated with Th1/Th17 immune response in PV patients. Finally, these data are important for better understanding of the immune aspects that control disease outcome, and they may also provide important information about why patients develop autoantibodies against desmogleins.

## 1. Introduction

Pemphigus vulgaris (PV) is a chronic autoimmune blistering disease affecting both males and females worldwide, which, in some cases, can be fatal. The overall incidence rate of PV ranges from 0.1 to 3.2 cases per100,000 inhabitants/year [1], and it seems to be more frequently reported in Ashkenazi Jews than in individuals from other ethnic groups [2]. Histologically, the disease is characterized primarily by the presence of IgG4 antibodies against desmoglein-3, a cadherin-like transmembrane glycoprotein that mediates cell-cell adhesion in keratinocytes of the epidermis and mucous membranes. The presence of these autoantibodies induces acantholysis (loss of cohesion) of keratinocytes in the suprabasal spinous layer, leading to the formation of skin and mucous membrane lesions. In addition to the humoral immune response characterized by the presence of autoantibodies and chemokines [3, 4], pro- and anti-inflammatory cytokines [5] and cell-mediated immune responses [6, 7] may also be involved in the pathogenesis of the disease, and they can be measured both locally and systemically.

Current knowledge on the role of chemokines and cytokines in the pathogenesis of PV is limited. So far, only two studies with opposing results have been published. In the first study, interferon- (IFN-) gamma-inducible protein-10 (IP-10), monokine induced by IFN-gamma (MIG), macrophage inflammatory protein- (MIP-) 1alpha, MIP-1beta, RANTES, eotaxin, monocyte chemoattractant protein- (MCP-) 1, MCP-2, MCP-3, and Growth-regulated oncogene-alpha (GRO-*α*) were unchanged in the serum of PV patients [4]. In the second study, MIG, thymus and activation regulated chemokine (TARC), and macrophage derived chemokine (MDC) were higher in PV patients than in healthy controls [3].

Unlike the results for chemokines, there are numbers of findings demonstrating the role of cytokines in the etiopathogenesis of PV. In the first study, D'Auria et al. (1997) evaluated cytokine levels in the serum of PV patients [8]. These authors showed that only 2 cytokines (IL-6 and TNF-*α*) out of 13 (IL-1*β*, IL-2, IL-4, IL-5, IL-6, IL-7, IL-8, IL-10, IL-11, IL-12, IFN-*γ*, TNF-*α*, and TGF-*β*) were increased in PV patients. Some years later, in addition to the correlation of increased levels of IL-6 and TNF-*α* in the pathogenesis of PV, higher levels of IL-8, IL1-*β*, and IL-15 were also found in the serum of PV patients, whereas the levels of IL-1*β* and IL-22 were lower than in controls [9–15]. Additionally, increased levels of cytokines IL-10 and/or IL-4 were observed in the blister fluid and serum of PV patients [7, 16]. These individuals had decreased levels of Th1 cytokines, IL-2, and IFN-*γ*, and this effect seems to be due to an increased production of IL-4 and IL-10, which suppresses the expansion and activation of Th1 cells [7]. Furthermore, data published by Satyam et al. [7] regarding the levels of IFN-*γ* and IL-10 were in disagreement with other studies that demonstrated undetected levels of IL-10 and moderately increased levels of IFN-*γ*, despite not being statistically significant [11, 17]. More recently, it was shown that IL-17-producing T-helper cells could also be involved in the initiation and maintenance of the disease. The Th17 pattern, in this case, seemed to be dependent on the presence of IL-23 produced by macrophages and dendritic cells in injured tissues [18].

In this conflicting scenario, it is reasonable to assume the importance of elucidating the role of chemokines and cytokines in the immunopathogenesis of PV. Therefore, in this study, we aimed to evaluate serum molecules that are associated with the different patterns of immune response in PV patients in an endemic area. Finally, we tried to clearly explain how these individuals systemically respond to tissue damage in the skin and mucosal membranes.

## 2. Materials and Methods

### 2.1. Patients

The study was conducted with patients from a hospital for blistering diseases located in Uberaba, in the state of Minas Gerais, Brazil, from January 2013 to December 2015. Peripheral blood serum samples were obtained from 20 healthy subjects and 20 patients with PV. Individuals who were part of the control group reported no autoimmune diseases, cancer, infectious diseases, disseminated inflammation, and/or allergies, nor were they related to the PV patients included in this study. The PV patients had clinical, pathological, and/or serological diagnosis and, depending on the patient, they had different degrees of skin involvement. Moreover, all patients included in this study were under pharmacological treatment to control the disease (Table 1) and were kept under specialized medical care. The extension of cutaneous damage was assessed according to Wallace's Rule of Nines [19]. All participants indicated their willingness to participate in the study by signing Informed Consent Form. The study was conducted in accordance with the Declaration of Helsinki, and the protocol was approved by the Ethics Committee of Federal University of Triângulo Mineiro under protocol number 1.300.898.

### 2.2. Distribution of Gender, Age, Ethnicity, Place of Residence, and Medical Treatment in PV Patients

All patients and controls were grouped according to gender, age, ethnicity, and place of residence. The control group consisted of 20 subjects, 7 males and 13 females, aged 35.38 ± 14.24 (median ± SD). When controls were grouped based on skin phenotype, 60% of these individuals were characterized as white, 20% as black, and 20% as biracial. Regarding the place of residence of the control subjects, 55% lived in urban areas, 5% lived in rural areas, and 40% lived in urban areas but had frequent contact with rural areas (Table 1). On the other hand, PV patients consisted of 20 subjects, 5 males and 15 females, aged 42.1 ± 15.68 (median ± SD). When these subjects were grouped based on skin phenotype, 55% of them were characterized as white, 10% were black, and 35% were biracial. As for the place of residence of PV patients, 30% lived in urban areas, 15% lived in rural areas, and 55% lived in urban areas but had frequent contact with rural areas (Table 1). At the time of blood collection, all patients were being treated with glucocorticoids (GCs); prednisone and dapsone were the most commonly used, at doses of 5–60 mg/day and 80–150 mg/day, respectively. The duration of therapy using GCs ranged from 6 months to 9 years, and cutaneous involvement from no skin lesions ranged up to 99% of cutaneous surface. The lesions were found in the skin, in the oral cavity, and near the genital mucosa (Table 2).

### 2.3. Chemokine and Cytokine Production

Cytometric Bead Array (CBA) (BD Bioscience, San Jose, CA, USA) was used for multiple simultaneous detection of human cytokines TNF-*α*, IFN-*γ*, TGF-*β*, IL-4, IL-6, IL-9, IL-10, and IL-17A and chemokines CCL-5 (RANTES), CXCL8 (IL-8), and CCL3 (MIP-1*α*). The production of cytokines IL-1*β*, IL-5, IL-12 IL-13, IL-15, IL-22, IL-23, IL-33, and IP-10 and of chemokines MCP-1 and eotaxin was assessed in the serum using the enzyme-linked immunosorbent assay (ELISA) (R&D Systems®, San Diego, CA, USA), according to the manufacturer's instructions.

### 2.4. Data Analysis and Statistics

Statistical analyses were performed using the Graphpad Prism software (Software 6.0, La Jolla, CA, USA). The* D'Agostino-Pearson* test was used to assess normality for all variables. Mann–Whitney test for nonparametric samples was used to compare cytokine and chemokine levels in the serum of healthy volunteers and PV patients. The Spearman correlation test was used to evaluate the strength of association between the variables. The results were expressed as the median ± SD (standard deviation), and the differences were considered significant when *p* < 0.05.

## 3. Results

### 3.1. Th1/Th2/Th17/Th9/Th22/Treg Cytokine Patterns in PV Patients

**Figure 1:**
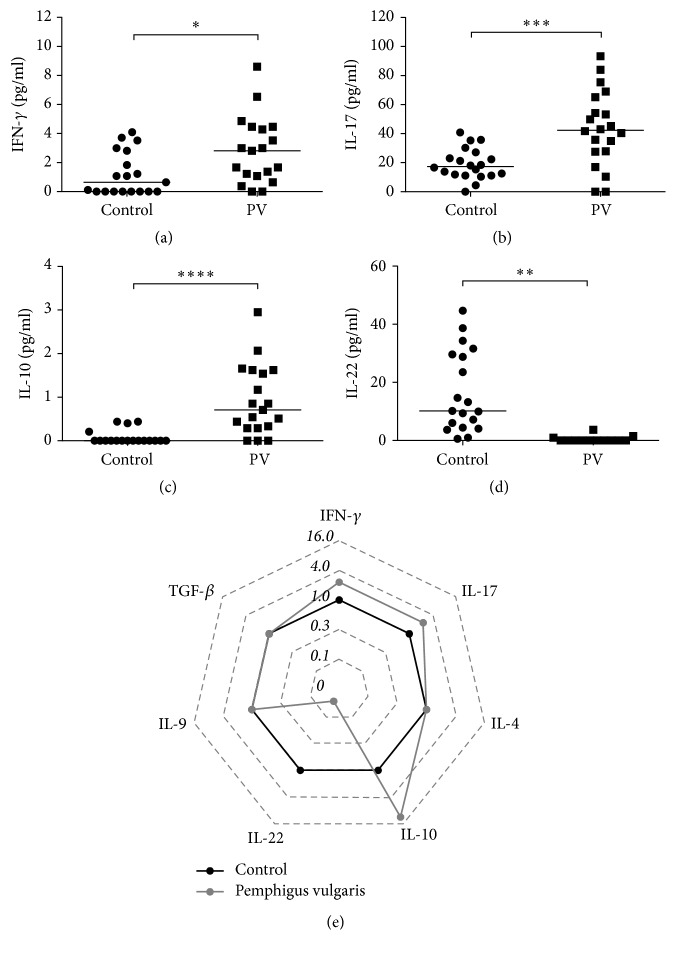
Serum profile of T cell-derived cytokines in Pemphigus vulgaris. Levels of (a) IFN-*γ*, (b) IL-17, (c) IL-10, and (d) IL-22 in Healthy Donors (⬤) and Pemphigus vulgaris patients (■). Error bars represent median ± SD. ^*∗*^*p* < 0.05; ^*∗∗*^*p* < 0.01; ^*∗∗∗*^*p* < 0.001; ^*∗∗∗∗*^*p* < 0.0001; Mann–Whitney test. (e) Radar plot representation of serum T cell-derived cytokine profile. The lines highlight the fold change in cytokine levels in Pemphigus vulgaris patients (gray line) in relation to Healthy Donors (black line). Data were obtained by calculating the ratio between the median concentration of each cytokine in the Pemphigus vulgaris group and in the healthy group.

First, given the importance of understanding the impact of different T-helper (Th) cytokines on disease outcome, Th1, Th2, Th17, Th22, Treg, and Th9 cytokine profiles were analyzed in PV patients and controls. Our results showed higher serum levels of Th1 (IFN-*γ*) and Th17 (IL-17) cytokines in patients with PV than in controls (Figures 1(a) and 1(b)). IL-22, which is released by both Th17 and Th22 cells, was decreased in these patients (Figures 1(d) and 1(e)). Furthermore, a significant increase in the production of IL-10 in the serum was observed in PV patients, which suggests an attempt of diseased patients to counter the inflammatory milieu by producing an anti-inflammatory cytokine (Figures 1(c) and 1(e)). Nonetheless, no differences were detected in the production of IL-4, IL-9, and TGF-*β* between control subjects and PV patients (data not shown).

### 3.2. Other Inflammatory Cytokines in PV Patients

**Figure 2:**
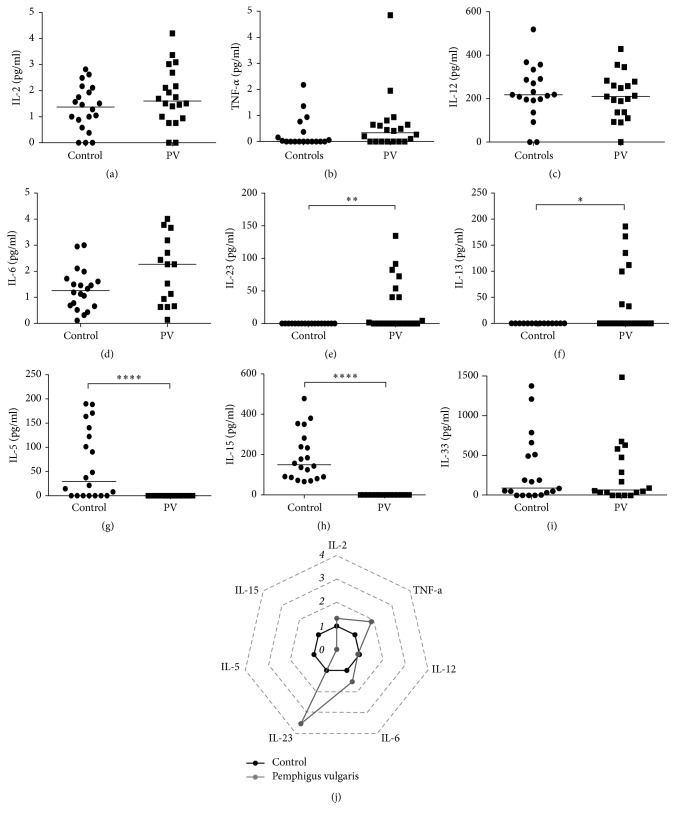
Serum profile of other proinflammatory cytokines in Pemphigus vulgaris. Levels of (a) IL-2, (b) TNF-*α*, (c) IL-12, (d) IL-6, (e) IL-23, (f) IL-13, (g) IL-5, (h) IL-15, and (i) IL-33 in Healthy Donors (⬤) and Pemphigus vulgaris patients (■). Error bars represent median ± SD. ^*∗*^*p* < 0.05; ^*∗∗*^*p* < 0.01; ^*∗∗∗∗*^*p* < 0.0001; Mann–Whitney test. (j) Radar plot representation of serum proinflammatory cytokine profile. The lines highlight the fold change in cytokine levels in Pemphigus vulgaris patients (gray line) in relation to Healthy Donors (black line). Data were obtained by calculating the ratio between the median concentration of each cytokine in the Pemphigus vulgaris group and in the healthy group.

The induction of one or more patterns of immune response leads to the production of pro- or anti-inflammatory cytokines. As the most induced cytokines were produced by Th1 and Th17 cells, we evaluated whether inflammatory cytokines resulting from or inducing these immune responses would be changed in patients with PV. Thus, the levels of cytokines IL-1, IL-2, IL-6, TNF-*α*, IL-12, IL-15, and IL-23 were evaluated. Moreover, the role of proinflammatory cytokines induced by Th2 immune response was also investigated. The production of IL-2, TNF-*α*, and IL-6 showed an increasing trend even though no statistically significant difference was observed (Figures 2(a), 2(b), and 2(d), respectively, and radar plot 2I). The production of IL-1 and IL-12 remained unchanged in our study (Figure 2(c)), and the production of IL-15 was suppressed and statistically lower in patients with PV than in control group patients (Figures 2(h) and 2(i)). The production of IL-23, either participating in the induction of or produced by Th17 cells, was increased in patients with PV (Figures 2(e) and 2(i)). The production of IL-5 and IL-13 showed contradictory results: the production of IL-13 was increased in patients with PV, whereas the production of IL-5 was decreased (Figures 2(f) and 2(g)). Thus, the results showed a clear relationship between the pathogenesis of PV and mixed Th1/Th17 immune response.

### 3.3. Chemokine Production in PV Patients

**Figure 3:**
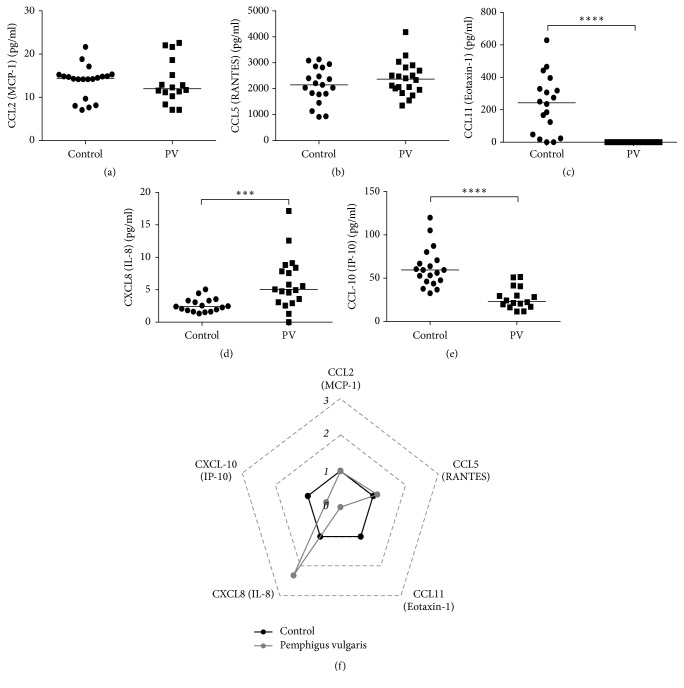
Serum profile of chemokines in Pemphigus vulgaris. Levels of (a) CCL-2/MCP-1, (b) CCL5/RANTES, (c) CCL11/Eotaxin, (d) CXCL8/IL-8, and (e) CCL10/IP-10 in Healthy Donors (⬤) and Pemphigus vulgaris patients (■). Error bars represent median ± SD. ^*∗∗∗*^*p* < 0.001; ^*∗∗∗∗*^*p* < 0.0001; Mann–Whitney test. (f) Radar plot representation of serum chemokine pattern. The lines highlight the fold change in cytokine levels in Pemphigus vulgaris patients (gray line) in relation to controls (black line). Data were obtained by calculating the ratio between the median concentration of each cytokine in the Pemphigus vulgaris group and in the control group.

In addition to the role of cytokines as important markers during the immune response, chemokines constitute another relevant source of information. As the production of Th1- and Th17-related cytokines was consistent among PV patients, we aimed to assess whether this immune profile would also be influenced by the production of chemokines. Our results showed no differences for the production of the Th1-related chemokines MIP-1*α* (CCL3) and MCP-1 (CCL2) between PV patients and their control counterparts (Figures 3(a) and 3(b), resp.). In comparison with controls, PV patients showed slightly increased levels of RANTES (CCL5) (Figure 3(f)). Furthermore, the chemokine IL-8 (CXCL8), which is associated with Th17 response, was also increased (Figures 3(d) and 3(f)) in individuals with PV, whereas the production of chemokines Th2 and IP-10 (CXCL-10), as well as of the CC chemokine eotaxin-1 (CCL11), was decreased in PV patients in comparison with controls (Figures 3(c) and 3(e), resp.).

### 3.4. Skin Lesions in Pemphigus Vulgaris Are Associated with Higher Levels of IL-6 and CXCL8

**Figure 4:**
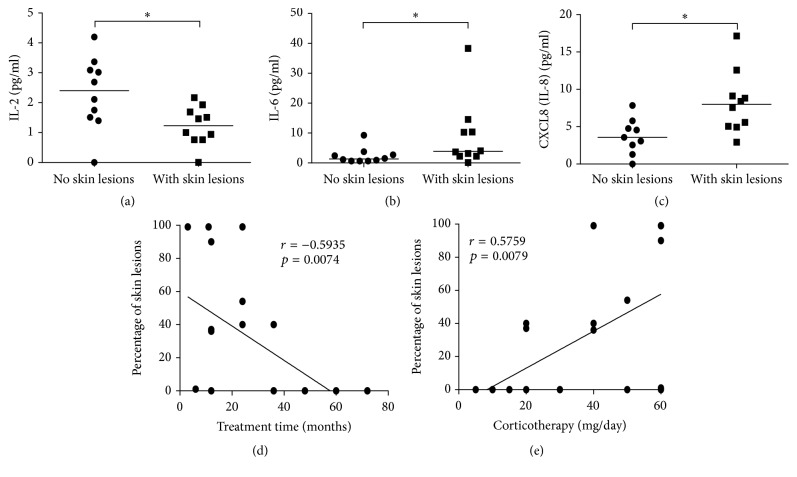
Skin lesions in Pemphigus vulgaris are associated with higher levels of IL-6 and CXCL8. Levels of (a) IL-2, (b) IL-6, and (c) CXL8 (IL-8) in Pemphigus vulgaris (PV) patients with no skin lesions (⬤) and PV patients with skin lesions (■). (d) Correlation between the frequency of lesions and time of treatment and (e) correlation between the frequency of skin lesions and dosage of glucocorticoids used to treat PV patients. Error bars represent median ± SD. ^*∗*^*p* < 0.05, Mann–Whitney test was used in (a–c). The Spearman correlation test was used to evaluate the strength of association between the variables (d and e).

As Th1- and Th17-related cytokines and chemokines have been correlated to the pathogenesis of PV, we aimed to evaluate whether this profile was also associated with disease activity. Because skin lesions characterize PV activity and are often controlled by immunosuppressive drugs, such as glucocorticoids, we evaluated the production of cytokines and chemokines in PV patients with or without skin lesions. Therefore, PV patients were subcategorized into patients without skin lesions (*n* = 10) or into patients with skin lesions (*n* = 10). The latter were directly associated with higher levels of IL-6 and CXCL8 (Figures 4(b) and 4(c), resp.), as well as with reduced levels of IL-2 (Figure 4(a)), when compared with the former. However, no differences were observed in the levels of the other chemokines and cytokines tested in this study (data not shown). Furthermore, there was a negative correlation between the impact of treatment time and the development of skin lesions (Figure 4(d)), but also a positive correlation between the need to use higher doses of glucocorticoids and treatment of a wide variety of skin lesions (Figure 4(e)). Overall, these data reinforce the roles of Th1 and Th17 chemokines and cytokines in the activity and worsening of PV. Furthermore, our results highlighted the need to use higher doses of immunosuppressive drugs in order to limit disease activity and skin lesion formation.

## 4. Discussion

Over the last two decades, some studies showed that cytokine and chemokine production in patients with* Pemphigus vulgaris* had changed. Nevertheless, these molecules were investigated in a fragmented, limited fashion, and their role in the pathogenesis of PV was not fully elucidated by these authors. In our study, the plasma levels of over 15 cytokines and 6 chemokines were evaluated in PV patients. The results pointed to a role of a mixed Th1/Th17 response in the pathogenesis of PV and to a positive correlation of these T helper cells with the production of other cytokines and chemokines in the serum. To our knowledge, this is the most complete and extensive study consistently addressing cytokine/chemokine profiles in PV patients.

The median age of PV patients was 40 years old with a female predominance (female : male = 3 : 1). The majority of patients were white or biracial (90%). The current study also revealed that the number of patients from an urban area or from an urban area with frequent contact with rural areas was higher than those living in a rural area. Female predominance is in accordance with a previous study that demonstrated the same distribution between genders [3]. On the other hand, our study showed intriguing results regarding the places of residence of PV patients, since most of them had no contact with rural areas. This observation is important because autoimmune blistering diseases, such as Pemphigus foliaceus, have been associated with patients from rural areas [20], particularly in Brazil, where the study was performed. Indeed, further studies with a larger number of patients must be performed in order to confirm or refute these findings. Ethnicity results were also significant in this study, since most patients diagnosed with PV were white. In other words, our findings are in accordance with the literature regarding a higher incidence in white population, such as Ashkenazi Jews, when compared to other ethnic groups.

PV patients had increased serum levels of IFN-*γ* and IL-17, which are cytokines associated with Th1 and Th17 immune responses, respectively. The increased levels of IFN-*γ* and IL-17 are in accordance with some previous studies [7, 11, 18], which reinforces our observation that PV induces a mixed Th1/Th17 immune response. The production of IL-23, which can be involved in the differentiation of Th17 cells, was also increased in our study. The higher levels of IL-23, accompanied by increased levels of IL-6, may have resulted in the increased production of IL-17, thus promoting the differentiation of Th17 cells, which probably contributed to the pathogenesis of PV. IL-22, a cytokine produced by both Th17 and Th22 cells, was reduced in our study. This is in accordance with earlier studies [15], suggesting that Th22 phenotype is not systemically induced in PV patients.

IL-10 production was also increased in PV patients in our study, which suggest that the inflammation triggered by Th1/Th17 immune response may have induced a counter regulatory response in an attempt to restore the immune balance and constrain the inflammation. Accordingly, another study also showed that IL-10 is increased in PV patients [16]. However, a study conducted by D'Auria et al. showed no differences in IL-10 levels between PV patients and their control counterparts [8]. On the other hand, IL-10 may be directly involved in the pathogenesis of PV due to its role in B-cell maturation and in the production of IgG4 autoantibodies (reviewed by [21]).

The production of Th2-related cytokines is controversial and requires a better understanding of PV pathogenesis. The levels of IL-5 were found to be decreased, whereas the levels of IL-13 were increased, but the levels of IL-4 and IL-33 remained unchanged. Therefore, it is possible to postulate that Th2-inflammatory immune response is not induced in PV patients.

As cytokines associated with Th1, Th2, and Th17 immune responses were detected in PV patients, we also addressed the role of chemokines associated with these immune profiles in the pathogenesis of PV in this study. The results showed that IL-8 was increased in PV patients, whereas eotaxin-1 and IP-10 were reduced. Despite inhibition of IP-10, our results are in accordance with other studies, in which Th1-chemokines (MCP-1, MIP-1*α*, and RANTES) remained unchanged in the serum of PV patients [4]. Furthermore, the induction of Th1 response demonstrated in our study may be supported by the induction of MIG in PV patients [3]. The inhibition of eotaxin may be associated with the reduction of IL-5 observed in our study. The latter works together with the former to induce eosinophil migration and consequent tissue damage [22]. As eosinophils are frequently involved in skin diseases, including Pemphigus vulgaris, we postulate that this process is inhibited by induction of immunomodulatory mechanisms in PV patients, or that such inhibition may even occur due to the direct effect of glucocorticoid therapy in these patients. On the other hand, the chemokine IL-8 was increased in the serum of PV patients, which is in disagreement with a previous study that demonstrated no changes in this chemokine in PV patients [8]. Taken together, the positive association between skin lesions, Th1- and Th17-related cytokines and chemokines, and disease activity further supports the role of these immune responses in the pathogenesis of PV. The discrepancies regarding cytokine and chemokine levels in distinct studies might be partly due to the different geographical areas, disease activity, and treatment type and exposure in each case.

## 5. Conclusion

To our knowledge, this is the most thorough and comprehensive study on the effects of cytokines and chemokines in the pathogenesis of Pemphigus vulgaris. Furthermore, our results reinforce the roles played by Th1 and Th17 cytokines in the modulation of the immune response in PV patients. In conclusion, we believe that this study may provide a better understanding of the cytokines and chemokines involved in disease outcome or of the development of autoantibodies against desmoglein.

## Figures and Tables

**Table 1:** Clinical findings in controls and in patients with Pemphigus vulgaris (PV).

	Controls	PV patients
Age (median ± SD)	35.38 ± 14.24	41.47 ± 15.82
Gender	7 M/13 F	5 M/15 F
Skin color:		
Biracial	4	8
White	12	11
Black	4	2
Living areas:		
Urban area	11	6
Urban and rural areas	8	11
Rural area	1	3

SD = standard deviation; M = males; F = females.

**Table 2:** Age, cutaneous involvement, and treatment used by Pemphigus vulgaris patients.

	Gender	Age (years)	Cutaneous involvement	Treatment period	Concomitant medications (dosage)
(1)	Female	43	No lesions	4 years	Prednisone (5 mg), dapsone (100 mg)
(2)	Male	64	Mild hyperpigmentation	9 years	Dapsone (50 mg)
(3)	Female	21	99% hyperpigmentation	3 years	Prednisone (10 mg), dapsone (100 mg), vitamin D
(4)	Female	38	99% hyperpigmentation	3 years	Prednisone (15 mg), dapsone (150 mg), vitamin D
(5)	Female	68	37% erythema	1 year	Prednisone (20 mg)
(6)	Female	53	9% hyperpigmentation	1 year	Predisim (3 mg), azathioprine
(7)	Female	48	No lesions	1 year	Prednisone (30 mg), Dapsone (100 mg)
(8)	Female	35	No lesions	5 years	Prednisone (20 mg), dapsone (80 mg)
(9)	Female	12	99% hyperpigmentation	4 months	Prednisone (20 mg)
(10)	Female	47	90% hyperpigmentation and erythema	6 years	Prednisone (20 mg)
(11)	Male	16	36% hyperpigmentation	3 years	Prednisone (20 mg)
(12)	Male	26	Lesions in granulation phase	2 years	Cyclosporine (250 mg)
(13)	Female	48	40% granulomatous lesions	3 years	Prednisone (20 mg), dapsone (100 mg)
(14)	Male	63	Oral lesions and bruises on segments	6 months	Prednisone (60 mg)
(15)	Male	28	90% active lesions and erythema	1 year	Prednisone (60 mg), dapsone (100 mg)
(16)	Female	46	99% acute lesions	3 months	Prednisone (60 mg), dapsone (100 mg)
(17)	Female	ni	99% acute lesions	11 months	Prednisone (60 mg)
(18)	Female	47	99% acute lesions and hyperpigmentation	2 years	
(19)	Female	50	54% hyperpigmentation and erythema	2 years	Prednisone (50 mg), Dapsone (100 mg), levothyroxine
(20)	Female	35	36% granulomatous lesions		Prednisone (40 mg), dapsone (100 mg)
